# Reslizumab and Eosinophilic Asthma: One Step Closer to Precision Medicine?

**DOI:** 10.3389/fimmu.2017.00242

**Published:** 2017-03-10

**Authors:** Gilda Varricchi, Gianenrico Senna, Stefania Loffredo, Diego Bagnasco, Matteo Ferrando, Giorgio Walter Canonica

**Affiliations:** ^1^Division of Clinical Immunology and Allergy, Department of Translational Medical Sciences, School of Medicine, University of Naples Federico II, Naples, Italy; ^2^Center for Basic and Clinical Immunology Research (CISI), University of Naples Federico II, Naples, Italy; ^3^Asthma Center and Allergy Unit, Verona University, General Hospital, Verona, Italy; ^4^Allergy and Respiratory Diseases, DIMI Department of Internal Medicine, IRCCS AOU San Martino-IST, University of Genova, Genova, Italy; ^5^Personalized Medicine Clinic Asthma and Allergy Humanitas Clinical and Research Center, Department of Biomedical Science, Humanitas University, Rozzano, Milano, Italy

**Keywords:** anti-IL-5, asthma, eosinophils, interleukin-5, IL-5 receptor, reslizumab

## Abstract

Human eosinophils represent approximately 1% of peripheral blood leukocytes. However, these cells have the propensity to leave the blood stream and migrate into inflamed tissues. Eosinophilic inflammation is present in a significant proportion of patients with severe asthma. Asthma is a chronic inflammatory disorder that affects more than 315 million people worldwide, with 10% having severe uncontrolled disease. Although the majority of patients can be efficiently treated, severe asthmatics continue to be uncontrolled and are at risk of exacerbations and even death. Interleukin-5 (IL-5) plays a fundamental role in eosinophil differentiation, maturation, activation and inhibition of apoptosis. Therefore, targeting IL-5 is an appealing approach to the treatment of patients with severe eosinophilic asthma. Reslizumab, a humanized anti-IL-5 monoclonal antibody, binds with high affinity to amino acids 89–92 of IL-5 that are critical for binding to IL-5 receptor α. Two phase III studies have demonstrated that reslizumab administration in adult patients with severe asthma and eosinophilia (≥400 cells/μL) improved lung function, asthma control, and symptoms. Thus, the use of blood eosinophils as a baseline biomarker could help to select patients with severe uncontrolled asthma who are likely to achieve benefits in asthma control with reslizumab. In conclusion, targeted therapy with reslizumab represents one step closer to precision medicine in patients with severe eosinophilic asthma.

## Introduction

Bronchial asthma is a chronic heterogeneous inflammatory disorder characterized by recurrent symptoms of reversible airflow obstruction, bronchial hyperresponsiveness, and airway inflammation (1, 2). It has been estimated that asthma affects more than 315 million people worldwide, with approximately 10% having severe or uncontrolled asthma (3, 4). In addition, the worldwide prevalence of asthma continues to increase and is projected to reach more than 400 million by 2020 (5). Importantly, approximately 250,000 deaths can be attributed to asthma each year, making it a severe chronic lung disorder (6).

Asthma’s manifestations can range from mild to very severe. The majority of patients can be efficiently treated with different drugs administered orally and/or by specific devices [e.g., inhaled glucocorticoids (ICS)]. Patients with severe asthma require treatment with high-dosage ICS or systemic glucocorticoids plus long-acting β_2_-agonists (GINA, accessed 2016). However, inhaled and systemic glucocorticoids can have multiple local (e.g., dysphonia and candidiasis) and systemic side effects (e.g., cataracts, osteoporosis, and adrenal suppression).

Despite the effectiveness of these treatments for most asthmatics, many patients continue to be uncontrolled and are at risk for severe asthma exacerbations or even death. These patients experience a high disease burden including recurrent exacerbations and hospital admissions (7). Finally, the cost of asthma treatment increases with disease severity (8).

The old concept that asthma represents a single disease has been replaced with the belief that it instead represents a heterogeneous mix of overlapping disorders that result from the interplay between multiple environmental factors (e.g., allergens, superallergens, viral and bacterial infections, etc.) that act in concert with hundreds of susceptibility genes (9–13).

Recently, it has been proposed that asthma can be classified according to two major endotypes. Endotype is a disease subtype defined by a distinct functional or pathological mechanism (14–16). “Th2-high” asthma is characterized by increased levels of type 2 inflammation, mainly mediated by eosinophils, mast cells, Th2 cells, group 2 innate lymphoid cells (ILC2s), and IgE-producing B lymphocytes (1). Patients with Th2-high asthma have eosinophilia and other signs of type 2 inflammation. By contrast, “Th2-low” asthma is less well characterized and probably represents a mix of multiple endotypes involving subgroups of patients (1, 17, 18).

Approximately 5–10% of asthmatic patients have severe asthma that is poorly controlled by drugs including high-dosage ICS and/or systemic glucocorticoids. The mechanisms of glucocorticoid subsensitivity/insensitivity in severe asthma are largely unknown (19). Several mechanisms have been proposed to explain glucocorticoid resistance of a subset of severe asthmatics (20–28). Glucocorticoid subsensitive asthmatic patients with eosinophilic are likely to benefit from anti-interleukin-5 (IL-5)/IL-5Rα therapies (19). As we move away from the traditional clinical description to include a multidimensional emphasis on cellular biology (endotypes and phenotypes), we increase our opportunity to provide targeted therapies, especially in more severe diseases (29). Accurate definition of asthma endotypes/phenotypes is critical in selecting targets for therapies, providing basis for targeted treatment of asthma (30).

## Eosinophils in Asthma

Approximately 5–10% of asthmatic patients have severe asthma that is poorly controlled by drugs including high-dosage of ICS and/or systemic glucocorticoids. The mechanisms of glucocorticoid subsensitivity or insensitivity in severe asthma are largely unknown (19).

Eosinophilic inflammation is present in a significant proportion of patients with severe asthma (31) and is associated with exacerbations and decreased lung function (32). Moreover, progressive increase in sputum and blood eosinophils is accompanied with poor pharmacological asthma control (33).

There is compelling evidence that eosinophils and their mediators are critical effectors to severe eosinophilic asthma and eosinophilic granulomatosis with polyangiitis (EGPA) (34, 35). EGPA is a systemic vasculitis frequently occurring in patients with severe asthma and eosinophilia. EGPA patients often have severe respiratory involvement that requires treatment with oral glucocorticoids (36).

**Figure 1:**
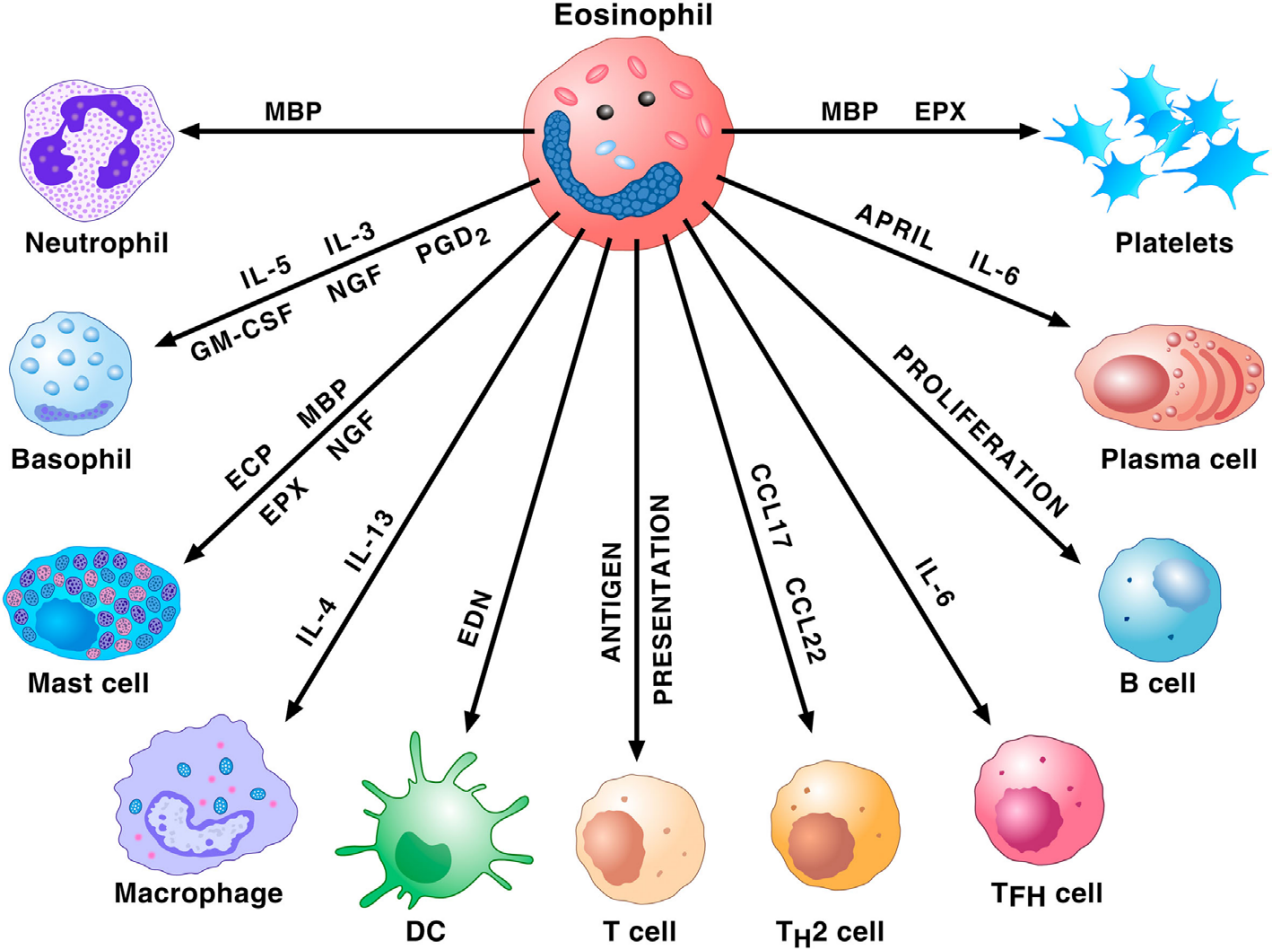
**Eosinophils and their mediators activate or modulate a plethora of cells of the innate and adaptive immune system**. Major basic protein (MBP) activates human neutrophils. Eosinophils prime/activate human basophils and mast cells through the release of cytokines [interleukin-5 (IL-5), interleukin-5 (IL-3), granulocyte-macrophage colony-stimulating (GM-CSF), NGF, and PGD_2_] or cationic proteins [eosinophil cationic protein (ECP), MBP, and eosinophil peroxidase (EPX)]. IL-4 and IL-13 favor M2 polarization of macrophages. EDN promotes the migration, maturation, and activation of dendritic cells (DCs). Eosinophils can act as antigen-presenting cells to initiate T cell responses and contribute to the recruitment of Th2 cells by producing the chemokines CCL17 and CCL22. These cells also favor T follicular helper (Tfh) cell maturation *via* the production of IL-6. Eosinophils promote B cell proliferation through an unknown mechanism. Eosinophils prime B cells and sustain plasma cells through the production of APRIL and IL-6. MBP and EPX induce platelet aggregation.

Due to their rarity (approximately 1% of peripheral blood leukocytes), eosinophils have been erroneously neglected for decades (37). During the last years, researchers of immediate hypersensitivity appreciated that these cells represent repositories of a wide spectrum of pro-inflammatory mediators such as several cationic proteins (major basic protein; eosinophil cationic protein; eosinophil peroxidase; and eosinophil-derived neurotoxin), cytokines/chemokines, and lipid mediators (35). Importantly, eosinophils have the capacity to adhere to activated endothelial cells, to leave the bloodstream and to concentrate at the site of allergic inflammation (38). These cells and their mediators are found in airway tissue and sputum of patients with asthma (39). In addition, human eosinophils play a major role in the modulation of the functions of a wide spectrum of cells of the innate and adaptive immune system, including subsets of lymphocytes, macrophages, mast cells, basophils, neutrophils, dendritic and plasma cells, and platelets (Figure 1).

## IL-5 and Its Receptor

**Figure 2:**
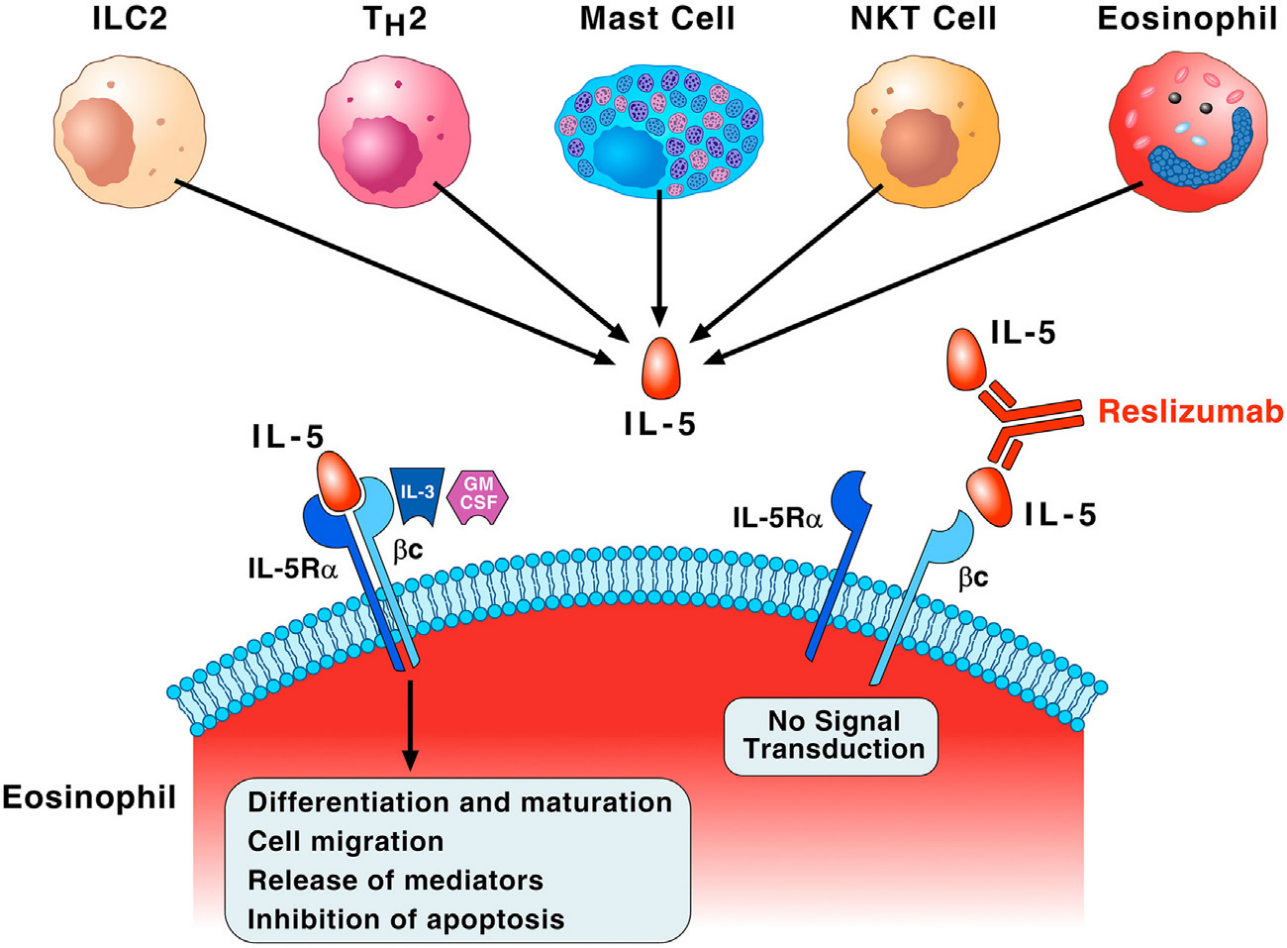
**Interleukin-5 (IL-5) plays a fundamental role in the growth, maturation, and activation of human eosinophils**. Group 2 innate lymphoid cell (ILC2), Th2 cells, mast cells, natural killer cells (NK T cells), and eosinophils themselves produce IL-5. This cytokine specifically binds to IL-5 receptor α (IL-5Rα) and recruits the βc subunit on the membrane of eosinophils. The βc subunit is the signal-transducing molecule shared with IL-3 and granulocyte-macrophage colony-stimulating (GM-CSF). This IL-5Rα/βc interaction leads to a series of biochemical events that control eosinophil differentiation and maturation in the bone marrow, cell migration to the site of allergic inflammation, the release of pro-inflammatory mediators, and inhibition of apoptosis. IL-3 and presumably GM-CSF, through binding to the βc of IL-5 receptor, can also function as eosinophil survival factors. Reslizumab binds with high affinity (*K*_d_ of approximately 50 pM) to amino acids 89–92 of human IL-5 that are critical for binding to IL-5Rα, resulting in inhibition of eosinophil maturation and activation.

Interleukin-5 is the most important growth, differentiation, and activating factor for human eosinophils (35). This cytokine is a dimeric protein with a 4-helix bundle motif, and it acts on target cells by binding to its specific IL-5 receptor (IL-5R), which consists of an IL-5 receptor α (IL-5Rα) subunit (IL-5Rα) and a common receptor β subunit (βc) (40, 41). IL-5Rα specifically binds IL-5 and induces the recruitment of βc to IL-5R (42). The βc subunit is a signal-transducing molecule shared with two receptors for monomeric cytokines, IL-3, and granulocyte-macrophage colony-stimulating (GM-CSF) (40). Figure 2 illustrates that IL-5 is mainly produced by ILC2, Th2 cells, mast cells, invariant natural killer cells (NK T cells), and eosinophils themselves (43). Although IL-5 is crucial for maturation and activation of human eosinophils (44), there is evidence that GM-CSF and IL-3 can function as eosinophil survival factors (45). Recent evidence indicates that IL-5, along with GM-CSF and IL-3, mediates eosinophil cellular survival by NF-Kb-induced Bcl-x_l_, which inhibits apoptosis (46). Interestingly, substantial levels of eosinophils remain after IL-5 neutralization or genetic deletion, suggesting that there are alternative pathways for promoting eosinophilia (47). Finally, IL-3 triggers prolonged signaling through activation of ribosomal protein S6 in human eosinophils providing new insight into the mechanisms underlined differential activation of eosinophils by IL-5 and IL-3 (48).

Whereas IL-5 is crucial for supporting mature eosinophils, the signals that support earlier eosinophil lineage events are less defined. Recent evidence indicates that IL-33 is required for basal eosinophil homeostasis (49) suggesting that this cytokine, through activation of ILC2 and their production of IL-5, plays a key role in promoting eosinophilopoiesis in response to allergen exposure (50).

Based on the previous observations, during the last decades targeting IL-5 or IL-5Rα appeared an interesting approach to the treatment of patients with severe eosinophilic asthma and hypereosinophilic-associated disorders (35, 51). Two humanized monoclonal antibodies directed against IL-5 [mepolizumab (proposed trade name Nucala; GSK) and reslizumab (proposed trade name Cinqair; Teva)] have provided an opportunity to investigate the role of this pathway in defining therapy of severe eosinophilic asthma (44, 51). Moreover, benralizumab (MedImmune/AstraZeneca), a humanized monoclonal antibody directed against the α-chain of the IL-5R, present on eosinophils and basophils (52), *demonstrated* efficacy and safety in adult patients with severe eosinophilic asthma (53, 54).

Reslizumab, previously known as Sch 55700, is a humanized, neutralizing anti-IL-5 antibody. Sch 55700 was humanized using complementary determining region drafting technology from a rat monoclonal antibody with a *K*_d_ of 53 pM against human IL-5 (55). The humanized antibody (reslizumab) retains the potency of the parent antibody, blocks IL-5R binding, and inhibits IL-5-induced cell proliferation (56). Reslizumab binds to a small region corresponding to amino acids 89–92 of IL-5 that are critical for binding to IL-5Rα (55) (Figure 2).

An initial multicenter study evaluated in a randomized, double-blind the effect of a single dose of i.v. reslizumab in a small group of severe asthmatics. The dose of 1 mg/kg produced a reduction in eosinophil counts but did not improve lung functions or symptom score (57). A subsequent multicenter, randomized, double-blind, and placebo-controlled study in poorly controlled asthmatics and sputum eosinophils ≥3% demonstrated that reslizumab (3 mg/kg i.v. every 4 weeks per four doses) reduced sputum eosinophils and improved airway function particularly in patients with nasal polyps (58). In two duplicated, multicenter, double-blind, parallel group, randomized, and placebo-controlled trials, a large number of patients were treated with reslizumab (3 mg/kg i.v. every 4 weeks per 13 doses). This monoclonal antibody reduced asthma exacerbations and improved FEV_1_ (59).

A phase III study further characterized the efficacy and safety of reslizumab (3 mg/kg i.v. every 4 weeks per four doses) in patients aged 12–75 years with asthma inadequately controlled by ICS and with a blood eosinophil count ≥400 cells/μL (60). Reslizumab improved lung function (FEV_1_), asthma control and symptoms, and quality of life (asthma control questionnaire and asthma quality of life questionnaire) and was well-tolerated. In another phase III study, the efficacy of reslizumab (3 mg/kg i.v. every 4 weeks per four doses) was evaluated in patients with poorly controlled asthma, particularly those with blood eosinophils ≤400 cells/µL (61). Interestingly, in the latter group of patients, clinically meaningful effects on lung functions (FEV1) and symptoms were not seen in patients unselected for baseline eosinophils.

The two latter studies emphasize the importance of selecting patients based on the number of eosinophils (≥400 μL) in peripheral blood. Thus, the use of blood eosinophils as a baseline biomarker could help to select patients who are likely to achieve more benefits in asthma control with reslizumab. Raised blood eosinophil count has recently been described as a useful biomarker to assess patients with eosinophilic asthma (53, 54, 60–64) and as a predictor of response to glucocorticoids (65). However, it should be emphasized that blood eosinophil counts do not accurately predict sputum eosinophils in severe asthmatics (66). Moreover, there is no correlation between sputum and blood eosinophil counts in severe glucocorticoid-dependent asthmatics (67).

In March 2016, the Food and Drug Administration concluded that reslizumab has an adequate safety profile and demonstrates the efficacy in treating severe eosinophilic asthma in adults. The approved dosage regimen is 3 mg/kg i.v. over 20–50 min every 4 weeks for patients aged ≥18 years.

## Concluding Remarks

Several studies have demonstrated that the i.v. administration of reslizumab is well-tolerated in adult patients with severe eosinophilic asthma up to 1 year. Recent evidences demonstrate that eosinophils play a role in cancer rejection (68, 69) and that several hematologic and tissue cancers can be associated with eosinophilia (70). In addition, it has been suggested that “targeted anti-eosinophilic strategies may unmask or even accelerate progression” of certain tumors in few patients with hypereosinophilic syndrome (71). Therefore, future surveillance and “real-life” studies will be needed to further confirm the safety of reslizumab in long-term treatment of patients with severe eosinophilic asthma. Recent studies have demonstrated the safety and efficacy of two monoclonal antibodies anti-IL-5—mepolizumab (63, 64) and reslizumab (59–61)—and of anti-IL-5 Ra (53, 54) in the treatment of adult patients with severe eosinophilic asthma. Different inclusion criteria (e.g., blood eosinophil count ≥300 vs ≥400/mL) and routes of administration (s.c. vs i.v.) of anti IL-5/anti-IL-5 Ra preclude a quantitative comparison of the results obtained in different trials.

A major advance in the development of a precision medicine approach for the treatment of severe asthma is the ability to select the appropriate patients. Ideally, patients should be selected by an easily measured biomarker. The studies with reslizumab treatment for severe eosinophilic asthma demonstrate that the blood eosinophil count (≥400 μL) appears closely associated with a clinical response in adult patients (59–61). Therefore, the age of precision medicine has arrived for the subset of severe asthmatics with an eosinophil-driven phenotype using anti-IL-5 therapy with reslizumab [(72) (posted online)].

Future studies should evaluate the safety and efficacy of reslizumab in children and in patients with other eosinophil-driven diseases. Preliminary studies in patients with nasal polyps (73) and in children and adolescents with eosinophilic esophagitis (74) demonstrated that reslizumab reduced tissue eosinophils without improvement in symptoms. Additional studies should investigate the optimal dose and strategy of reslizumab treatment in these and other eosinophil-driven diseases (e.g., atopic dermatitis and EGPA).

Future studies should also evaluate the outcome of patients with severe eosinophilic asthma after discontinuation of reslizumab therapy. A preliminary study reported that cessation of mepolizumab in patients with eosinophilic asthma resulted in a rapid increase of blood eosinophils followed by gradual increase in asthma symptoms and exacerbations (75). This interesting observation emphasizes the importance of maintaining suppression of eosinophilic inflammation in severe asthmatics.

Targeted therapy with reslizumab appears to be effective and safe in the treatment of adults with severe eosinophilic asthma and represents one step closer to precision medicine.

## Author Contributions

All authors listed have made substantial, direct, and intellectual contribution to the work and approved it for publication.

## Conflict of Interest Statement

The authors declare that the research was conducted in the absence of any commercial or financial relationships that could be construed as a potential conflict of interest.

## Abbreviations

ACQ, asthma control questionnaire; AQLQ, asthma quality of life questionnaire; ECP, eosinophil cationic protein; EDN, eosinophil-derived neurotoxin; EPX, eosinophil peroxidase; FDA, Food and Drug Agency; FEV_1_, forced expiratory volume in the 1st second; GM-CSF, granulocyte-macrophage colony-stimulating factor; ICS, inhaled glucocorticoids; ILC, innate lymphoid cells; IL-5, interleukin-5; IL-3, interleukin-3; LABA, long-acting β_2_-agonists; MBP, major basic protein; NK T cells, natural killer cells.
